# The Relationship Between Quality of Discharge Teaching and Oral Nutritional Supplementation Adherence in Postoperative Patients With Gastric Cancer: A Chain Mediated Role of Readiness for Hospital Discharge and Medication Beliefs

**DOI:** 10.1111/jocn.70029

**Published:** 2025-07-07

**Authors:** Fang Shen, Xiaoxue Chen, Hong Yu, Fang Xiao, Xiuhong Yuan, Jia Li

**Affiliations:** ^1^ Department of Gastric Surgery State Key Laboratory of Oncology in South China, Guangdong Provincial Clinical Research Center for Cancer, Sun Yat‐Sen University Cancer Center Guangzhou Guangdong China

**Keywords:** adherence, gastric cancer, medication beliefs, oral nutritional supplementation, quality of discharge teaching, readiness for hospital discharge

## Abstract

**Aim:**

We aimed to elucidate the underlying mechanisms influencing Oral nutritional supplementation (ONS) adherence in postoperative patients with gastric cancer (GC) by developing a structural equation model.

**Background:**

ONS represents a cost‐effective nutritional intervention for postoperative patients with GC, with its efficacy largely dependent on sustained patient adherence over time. However, the interrelationships among the quality of discharge teaching (QDT), readiness for hospital discharge (RHD), medication beliefs and adherence to ONS remain inadequately understood.

**Methods:**

A convenience sample of 505 postoperative patients with GC was recruited from January 1, 2023, to December 1, 2024, for a cross‐sectional survey conducted at a tertiary‐grade A specialised oncology hospital. The data of this study were subjected to descriptive analysis, Harman's one‐way analysis of variance, Pearson correlation analysis and mediation effect analysis.

**Reporting Method:**

The STROBE checklist was employed for reporting in the study.

**Results:**

Pearson correlation analyses revealed that all four variables were significantly interrelated. Structural equation modelling showed that medication beliefs had the strongest correlation with ONS adherence (*β* = 0.589), followed by readiness for hospital discharge (RHD) (*β* = 0.557) and quality of discharge teaching (QDT) (*β* = 0.523). The structural equation model demonstrated a robust overall fit.

**Conclusion:**

There was a significant chain mediation effect through RHD and medication beliefs. For the development of targeted intervention strategies to improve ONS adherence, future research should prioritise enhancing QDT, optimising RHD and strengthening patients' medication beliefs.

**Relevance to Clinical Practice:**

To help nurses and nursing managers formulate intervention measures to improve QDT, RHD, medication beliefs and ONS adherence in postoperative patients with GC.

**Summary**

- What does this paper contribute to the wider global clinical community?
  ○ This study found that for postoperative patients with gastric cancer (GC), the quality of discharge teaching (QDT) was relatively low and oral nutritional supplementation (ONS) adherence was poor.
  ○ This study found that readiness for hospital discharge (RHD) and medication beliefs acted as mediators in the relationship between the QDT and ONS adherence.
  ○ This study also provides a theoretical and practical direction for future research aimed at improving patients' QDT, RHD, medication beliefs and ONS adherence in postoperative patients with GC.

**Abbreviations**
BMQ: Beliefs about Medical Questionnaire
GC: gastric cancer
ONS: oral nutritional supplementation
ONSAS: Oral Nutritional Supplementation Adherence Scale
QDT: quality discharge teaching
QDTS: quality of Discharge Teaching Scale
RHD: readiness for Hospital Discharge
RHDS: readiness for Hospital Discharge Scale

## 1 Introduction

Gastric cancer (GC) is one of the most common malignant neoplasms in the digestive system, characterised by its high malignancy and poor prognosis. According to the 2020 global cancer statistics, there were approximately 1.089 million new GC cases and 769,000 related deaths globally, with China accounting for 43.9% and 48.6% of these figures, respectively (Global Cancer Observatory 2021; Sung et al. 2021). Malnutrition is a significant concern among patients, with a reported incidence ranging from 50% to 80% (Song et al. 2019). It is a key contributor to postoperative complications, prolonged hospitalisation, increased healthcare costs, and decreased overall survival (Kim et al. 2017; Minnella et al. 2018).

Oral nutritional supplementation (ONS) has been widely recommended as a cost‐effective therapy to address malnutrition and improve postoperative recovery (Muscaritoli et al. 2021; Wobith and Weimann 2021). However, its clinical effectiveness is heavily reliant on patient adherence. Adherence, defined as the degree to which patient behaviours align with medical advice (Kim et al. 2011), is critical because the benefits of ONS are dose‐dependent (Ferreira et al. 2025; Lidoriki et al. 2020). Despite clinical guidelines recommending ONS for at least 3–6 months post‐discharge, adherence remains poor. For instance, a recent study showed that only 30.59% of GC patients adhered to ONS for 12 weeks post‐discharge, with a sharp decline noted within the first 5 weeks (Su et al. 2024). This low adherence rate can be attributed to multiple factors. Many patients lack awareness of the importance of nutritional support, often reducing or stopping ONS due to misconceptions or side effects. Barriers include taste intolerance, gastrointestinal discomfort, cost, limited health literacy and lack of family or professional support (Liljeberg et al. 2024). These challenges indicate the need for a more comprehensive understanding of behavioural and system‐level influences on adherence.

High‐quality discharge teaching (QDT) emerges as a key intervention to improve adherence. Defined as individualised, comprehensive education that prepares patients and caregivers for self‐management post‐discharge, QDT has been shown to enhance patient outcomes and reduce readmissions by improving patient understanding of care instructions, enhancing clarity in follow‐up care, and preventing complications (Guan and Feng 2023; Hua et al. 2020; Oh et al. 2023). Evidence indicates that effective instructions can reduce avoidable readmissions by 64%, while clear follow‐up plans are associated with a 34% lower risk (Alyahya et al. 2016). QDT emphasises patient‐centredness by tailoring content to patients' health literacy, social context and emotional readiness. For example, practical strategies include the use of teach‐back methods, integrating caregivers in the teaching process, using culturally appropriate materials and reinforcing information through multimedia or follow‐up calls. When applied effectively, QDT not only enhances understanding but also builds patient confidence in continuing ONS independently.

However, the relationship between QDT and ONS adherence remains underexplored. Previous studies suggest a link between discharge education and nutritional compliance (Wang et al. 2023). Other studies have clarified the mechanisms or mediating factors involved in this relationship, especially in GC populations. One possible mediator is readiness for hospital discharge (RHD), a measure of a patient's perceived preparedness to manage care at home. RHD is influenced by the quality of education, emotional status and the presence of support systems (Mabire et al. 2019; Meng et al. 2020). Studies in chronic illness populations show that higher RHD predicts better adherence to post‐discharge regimens, including medication (Axelsson et al. 2020; Zhang et al. 2014).

Another influential factor is medication beliefs. Patients with stronger medication beliefs of ONS tend to have fewer concerns about its potential adverse effects and are more likely to adhere to the prescribed regimen, thereby improving overall compliance (Steinmann et al. 2022; Su et al. 2024). A proper understanding of the relationship between QDT, RHD, medication beliefs and ONS adherence can contribute to improving nutritional status in postoperative patients with GC.

According to Meleis's transition theory, transition is a complex process and outcome that arises from the dynamic interaction between individuals and their environment (Meleis et al. 2000). During this, patients require support from both their families and health providers to meet their comprehensive healthcare needs. This theory comprises four parts: the transition nature (including the types, patterns and attributes of transition), the transition conditions (including the factors that facilitate and impede transition), the response (involving the process and outcome indicators of transition), and the nursing therapy (being the intervention measures that affect the outcome of transition).

**FIGURE 1:**
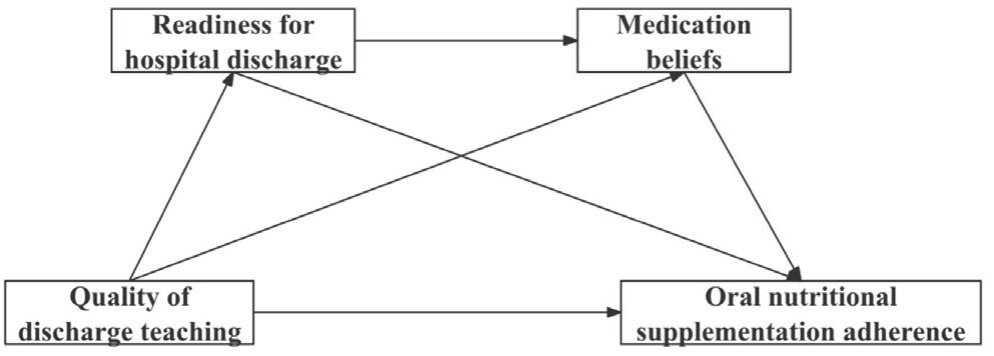
The hypothetical theoretical model.

In this study, the transition nature pertains to postoperative patients with GC experiencing a shift from hospitalisation to home or community settings; the transition conditions were derived from the demographic and clinical data of postoperative patients with GC; the QDT was categorised under nursing therapy; RHD and medication beliefs served as process indicators; and ONS adherence was identified as an outcome indicator. The transition theory provides a theoretical foundation for this study to elucidate the relationships among the QDT, RHD, medication beliefs and ONS adherence. The theoretical framework of this study is illustrated in Figure 1. The study tests four hypotheses: (1) QDT positively predicts ONS adherence; (2) RHD acts as a mediator between QDT and ONS adherence; (3) Medication beliefs act as a mediator between QDT and ONS adherence; (4) RHD and medication beliefs act as chained mediators between QDT and ONS adherence.

Although previous studies have explored factors influencing ONS adherence, most have examined variables in isolation and rarely within a theoretical framework. There is limited understanding of how discharge teaching, patient readiness and medication beliefs interact to shape adherence, particularly in the postoperative gastric cancer patients. This study addresses these deficiencies by constructing a structural equation model to examine a chain mediation pathway linking QDT, RHD and medication beliefs to ONS adherence. By integrating these factors into a cohesive model, our research offers both theoretical innovation and practical relevance for improving patient outcomes.

## 2 Materials and Methods

### 2.1 Participants

Convenience sampling was employed to recruit participants who had undergone surgery for GC in this study. Inclusion criteria were patients who were: (1) aged over 18–85 years (2) diagnosed with GC based on the Chinese Society of Clinical Oncology Clinical Guidelines for Gastric Cancer (Wang et al. 2024) and undergoing radical surgery; (3) with an expected survival period of more than 1 month; (4) postoperatively prescribed ONS in powered form, uniformly provided by the hospital during this study period; and (5) voluntary to participate and provide a signed informed consent form. The exclusion criteria were patients with (1) severe cardiovascular diseases; (2) severe mental illness that may impair cognitive function or compliance, including major depressive disorder, schizophrenia, bipolar disorder, or severe cognitive dysfunction; (3) difficulties in reading and writing; (4) a history of milk or protein drug allergy; and (5) an inability to cooperate with the investigators.

The sample size in this study was calculated using G*Power software (version 3.1.9.4), assuming an anticipated effect size of 0.15 (Faul et al. 2007). The significance level was set at the alpha level of 0.05, while the statistical power was set to 0.95. Ultimately, the sample size calculation determined that 472 participants were required in this study. The sample size was adjusted to prevent loss to follow‐up, missing data or unqualified samples.

Content validity was assessed by a panel consisting of three clinical experts in oncology nursing and two former patients who had experience with ONS. They reviewed the questionnaire items for relevance, clarity and comprehensiveness during the development phase.

### 2.2 Measurements

#### 2.2.1 General Information Questionnaire

The general information questionnaire, including information on socio‐demographic and health‐related data, was developed based on a comprehensive literature review and expert consultations. The general data of recruited patients, including age, sex, residence, education level, marital status, living arrangement, household income, medical insurance, chronic disease history, chemo‐radiotherapy history, Laurén classification and extent of gastric resection, were recorded.

#### 2.2.2 Oral Nutritional Supplementation Adherence Scale (ONSAS)

The English version of the Adherence Rating Scale, originally developed by Byerly et al. was translated and culturally adapted into Chinese (Byerly et al. 2008; Lai et al. 2016). Numerous studies have confirmed that the ONSAS could be utilised to assess ONS adherence in Chinese patients with gastroenteric tumours (Qin et al. 2022; Wan, Xue, et al. 2021). This scale comprises 6 items, each rated on a 1‐to‐5 scale. The total score ranges from 6 to 30 points. The cutoff score of 15 (indicating poor vs. good adherence) was referenced from Hou et al. (Hou et al. 2024). This threshold was used for descriptive purposes only, as the variable was modelled continuously. An example item is: ‘Did you take your oral nutritional supplements every day as prescribed?’ The Cronbach's *α* coefficient of this scale is 0.985 (Wang et al. 2016); in this study, the Cronbach's *α* coefficient is 0.836.

#### 2.2.3 Quality of Discharge Teaching Scale (QDTS)

The English version of the QDTS was originally developed by Weiss et al. (2007), and the Chinese version utilised in this study was translated and culturally adapted by Wang (Wang et al. 2016). This scale comprises three dimensions: teaching content needed (6 items), teaching content acquired (6 items), and teaching skills and effects (12 items), with a total of 24 items. An example item from the ‘teaching content needed’ dimension is: ‘How much information do you feel you needed about how to care for yourself at home?’. From the ‘teaching content acquired’ dimension: ‘How much information did you actually receive about how to take care of yourself at home?’. From the ‘teaching skills and effects’ dimension: ‘The nurse used understandable language during teaching.’

The total score is calculated as the average of the sum of item scores across the teaching content acquired and teaching skills and effects dimensions. QDTS scores range from 0 to 10, and a higher score characterises higher quality. The score bands (e.g., < 7 = inadequate, 7.0–7.9 = moderate, 8.0–8.9 = high, 9.0–10.0 = excellent) were derived from Weiss et al. (2014) and have been adopted in related structural equation modelling research on hysterectomy patients (Guan and Feng 2023). These were used to provide clinical interpretability but not for statistical categorization. The Cronbach's *α* coefficient for this scale was 0.924 (Wang et al. 2016); in this study, the Cronbach's *α* coefficient was 0.941.

#### 2.2.4 Readiness for Hospital Discharge Scale (RHDS)

The English version of the *RHDS* was developed by Weiss based on the Meleis's transition theory, and the Chinese version was translated and validated by Lin et al. in 2014 (Lin et al. 2014; Meleis et al. 2000). The scale includes three dimensions: personal status (three items), coping ability (five items), and expected support (four items), with a total of 12 items. An example item from the ‘personal status’ dimension is: ‘I feel physically ready to go home.’. From the ‘coping ability’ dimension: ‘I am confident I can take care of myself after discharge.’. From the ‘expected support’ dimension: ‘I expect to get support from my family when I get home.’

The total score of the scale is calculated as the average of the scores across the three dimensions, and higher scores indicate a greater level of readiness. The items are scored according to a 10‐point scale, where 0 indicates not well prepared and 10 indicates very well prepared. The classification thresholds for RHDS (0–7 = insufficient, 7–8 = moderate, 8–9 = high, 9–10 = excellent readiness) were based on Weiss et al. (2014) and have been adopted in related structural equation modelling research on hysterectomy patients (Guan and Feng 2023). Although used for reference, this study analysed RHDS scores as continuous variables. The Cronbach's *α* coefficient of this scale is 0.890 (Lin et al. 2014; Meleis et al. 2000); in this study, the Cronbach's *α* coefficient is 0.904.

#### 2.2.5 Beliefs About Medical Questionnaire (BMQ)

The BMQ was developed by Horne et al. in 1999, and the Chinese version used in this study was translated and revised by Feng et al. (Horne et al. 1999; Feng et al. 2019). The Chinese version of the BMQ can effectively evaluate the medication beliefs of patients with chronic disease, and it is applicable and suitable for postoperative patients with GC receiving ONS (Zhou et al. 2024). This scale comprises two dimensions: the perceived necessity of medication and concerns about medication, with each dimension containing five items, for a total of ten items. An example item from the ‘perceived necessity of medication’ dimension is: ‘Oral nutritional supplements play a crucial role in preventing the progression of my condition’. From the ‘concerns about medication’ dimension: ‘Sometimes I experience concerns regarding my reliance on oral nutritional supplements’.

Each item is scored on a 5‐point Likert scale, with scores ranging from 1 (strongly disagree) to 5 (strongly agree). The score for each of the two dimensions ranges from 5 to 25 points, and higher scores indicate a stronger level of belief in the corresponding dimension. The total score is calculated as the difference between the perceived necessity of medication score and the perceived concerns of medication score. The BMQ scoring formula (total = necessity to concern) and interpretation were based on the Chinese validation and application by Zhou et al. (Zhou et al. 2024). While no categorical cutoff was used in the analysis, the scoring range (−20 to +20) provides reference for the belief strength. The Cronbach's *α* coefficient of this scale is 0.770.

### 2.3 Data Collection and Quality Control

Before the formal investigation, two investigators received standardised training, including the investigation purpose, implementation methods, platform usage techniques and communication skills. Investigators were required to be familiar with the questionnaire content and use standardised instructions to explain the purpose, significance, methods and precautions of the investigation for respondents. After obtaining their signed electronic informed consent form, an online questionnaire was distributed via the Questionnaire Star Platform (Wenjuanxing, Changsha Ranxing Information Technology Co. Ltd). The questionnaire consisted of information on socio‐demographic and health‐related issues; QDT, RHD, and medication beliefs were collected on the day of discharge. ONSAS was collected one month post‐discharge, and patients usually revisited the hospital for review on this day.

The variables related to QDT, RHD and medication beliefs were collected on the day of discharge, when patients had confirmed their discharge arrangements. Adherence to ONS, however, was assessed 1 month post‐discharge, as prior studies have shown this to be the period when adherence is typically at its lowest (Su et al. 2024). Participants completed this part of the questionnaire during their follow‐up hospital review or via a follow‐up call if a review did not occur.

The investigation was conducted anonymously, with strict privacy of respondents' information. The time limit for completing the questionnaire was set to 20 min, and each account could only submit the questionnaire once to avoid duplication. In addition, due to the setting of the platform, questionnaires containing incomplete or missing items will not be permitted for submission. When patients could not complete the online questionnaire independently due to low literacy or the unfamiliar use of smartphones, the researcher would collect all the questionnaire data by telephone. Prior to data entry, two researchers who did not participate in the study reviewed and checked each questionnaire independently. Invalid questionnaires with too short an answering time, the same answer options or regular answers were excluded. Double‐checks of the manual data entry were employed by two researchers to minimise the errors and omissions, thereby ensuring data accuracy. The dataset is available in the Research Data Deposit public platform (www.researchdata.org.cn), with the approval number as RDDA2025124470.

### 2.4 Statistical Analysis

In this study, IBM SPSS Statistics 26 (SPSS for Windows, version 26, SPSS Inc., Chicago, Illinois, USA) and Amos version 24.0 (IBM Corp., Armonk, New York, USA) software were used to perform statistical analyses. Harman's single‐factor test was used to investigate whether there was significant common method bias in the data. All variables showed absolute skewness values less than 3 and absolute kurtosis values less than 8, indicating approximate normal distribution and justifying the use of parametric statistical methods (West et al. 1995). The counting data were expressed as numbers (*n*) and frequencies (%), whereas measurement data were presented as mean (*M*) and standard deviation (SD). Pearson correlation analysis was conducted to examine the relationships among the QDT, RHD, medication beliefs, and ONS adherence. Next, structural equation modelling was performed to test the structural relationships, and the Percentile Bootstrap method was conducted to validate the mediation effects. *p* < 0.05 indicated statistical significance. The 95% confidence intervals (CI) for the mediating effects were tested using bootstrap methods with 5000 bootstrap samples (Hayes 2017).

### 2.5 Ethical Considerations

This study was approved by the Ethics Committee of Sun Yat‐sen University Cancer Center (date 2022‐2‐25; Reference SL‐B2022‐091‐03), adhering to the STROBE statement guidelines for cross‐sectional studies (von Elm et al. 2007) and the Declaration of Helsinki.

## 3 Results

**FIGURE 2:**
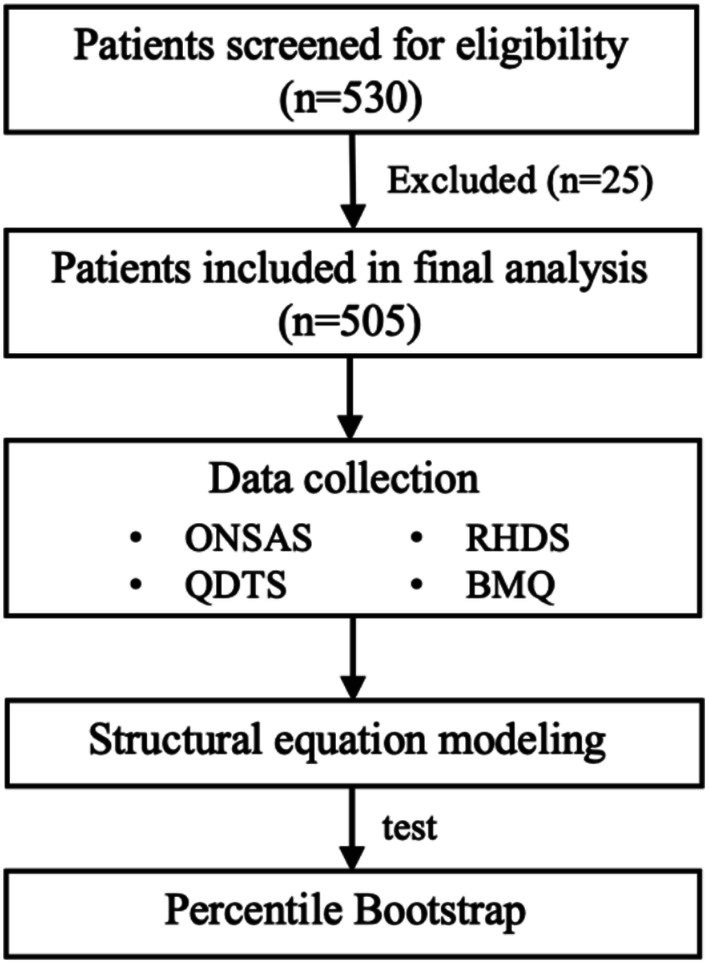
Flowchart of participant selection and data analysis procedure. BMQ, Beliefs about Medical Questionnaire; ONSAS, Oral Nutritional Supplementation Adherence Scale; QDTS, Quality of Discharge Teaching Scale; RHDS, Readiness for Hospital Discharge Scale.

In total, 530 postoperative patients with GC at discharge from the hospital were enrolled in this study. There were 25 patients lost to follow‐up due to loss of contact (*n* = 8), too short a response time (*n* = 7), refusal (*n* = 6), serious gastro‐intestinal side effects (*n* = 2), other serious adverse events (*n* = 1), and unplanned rehospitalisation (*n* = 1). Finally, 505 valid questionnaires were obtained, and the response rate was 95.3%. The sampling flowchart of this study is shown in Figure 2.

### 3.1 Common Method Bias

All data were collected from self‐reported responses in the questionnaire and may be subject to self‐report bias. To avoid potential common method variance, an unrotated exploratory factor analysis of all measures was carried out using Harman's single‐factor test. There were 6 common factors in total, each with an eigenvalue exceeding 1. With 34.81% of the total variation explained, the first common factor is below the 40% criterion proposed by Podsakoff et al. (Podsakoff et al. 2003). Thus, there was no serious common method bias with the data.

### 3.2 General Participant Information

This study recruited 296 males (58.6%) and 209 females (41.4%), and more than half lived in the towns (59.4%). The mean age of participants was 58.1 (SD = 11.9) years, and the majority were aged over 50 years. Most participants lived with their spouse or children (85.8%) and had health insurance (84.4%). 389 (77.0%) participants underwent partial gastrectomy, and 116 (23.0%) underwent laparoscopic surgery. Further detailed characteristics of the participant information are shown in Table 1.

**TABLE 1:** General characteristics of the study participants (*N* = 505).

Item	*n*	(%)
Age (years)		
< 50	103	20.4%
50–59	167	33.1%
≥ 60	235	46.5%
Sex		
Male	296	58.6%
Female	209	41.4%
Residence		
Village	205	40.6%
Towns	300	59.4%
Education level		
College or university	82	16.2%
Junior or high school	188	37.3%
Primary and below	235	46.5%
Marital status		
Bereaved of spouse	62	12.3%
Divorced	11	2.2%
Married	402	79.6%
Unmarried	30	5.9%
Living arrangement		
With spouse	195	38.6%
With children	238	47.2%
Alone	37	7.3%
Other	35	6.9%
Household income (yuan)		
< 1000	43	8.5%
1000–2999	91	18.0%
3000–4999	173	34.3%
≥ 5000	198	39.2%
Medical insurance		
Self‐financing	79	15.6%
Commercial/resident/employee medical insurance	246	48.7%
New rural cooperative medical scheme insurance	180	35.6%
Chronic disease history		
No	297	58.8%
Yes	208	41.2%
Chemo‐radiotherapy history		
No	310	61.4%
Yes	195	38.6%
Laurén classification		
Diffuse type	91	18.0%
Intestinal pattern	397	78.6%
Other	17	3.4%
Extent of gastric resection		
Partial gastrectomy	389	77.0%
Total gastrectomy	116	23.0%

### 3.3 Questionnaire Scores

The mean QDT, RHD, medication beliefs, and ONS adherence scores of postoperative patients with GC were 3.88 (SD = 2.05), 4.15 (SD = 2.12), −1.95 (SD = 10.14), and 13.70 (SD = 5.32), respectively. Table 2 illustrates the scores of other scale dimensions.

**TABLE 2:** Questionnaire scores (*N* = 505).

Variables	Theoretical value of score	Min	Max	Mean (SD)
QDT	(0, 10)	1	8	3.88 (2.05)
Teaching content acquired	(0, 10)	1	9	3.95 (2.23)
Teaching skills and effects	(0, 10)	0	8	3.85 (2.08)
RHD	(0, 10)	1	9	4.15 (2.12)
Personal status	(0, 10)	0	9	4.05 (2.39)
Coping ability	(0, 10)	0	9	4.16 (2.25)
Expected support	(0, 10)	0	9	4.21 (2.39)
Medication beliefs	(−20, 20)	−19	18	−1.95 (10.14)
Perceived necessity	(5, 25)	5	25	13.66 (5.40)
Perceived concerns	(5, 25)	5	25	15.61 (5.42)
ONS adherence	(6, 30)	6	30	13.70 (5.32)

Abbreviations: Max, maximum; Min, minimum; ONS, oral nutritional supplementation; QDT, quality of discharge teaching; RHD, readiness for hospital discharge; SD, standard deviation.

### 3.4 Correlation Analysis

Pearson correlation analysis showed that QDT was significantly positively correlated with RHD (*r* = 0.683, *p* < 0.010), medication beliefs (*r* = 0.618, *p* < 0.010), and ONS adherence (*r* = 0.523, *p* < 0.010). Medication beliefs were significantly positively correlated with RHD (*r* = 0.675, *p* < 0.010) and ONS adherence (*r* = 0.589, *p* < 0.010). There is a significant positive correlation between RHD and ONS adherence (*r* = 0.557, *p* < 0.010). The correlation coefficients between the variables are presented in Table 3.

**TABLE 3:** Correlation analysis of variables (*N* = 505).

	ONS adherence	QDT	RHD	Medication beliefs
ONS adherence	1			
QDT	0.523**	1		
RHD	0.557**	0.683**	1	
Medication beliefs	0.589**	0.618**	0.675**	1

Abbreviations: ONS, oral nutritional supplementation; QDT, quality of discharge teaching; RHD, readiness for hospital discharge.

** *p* < 0.010.

### 3.5 The Chain Mediating Regression Analysis

As shown in Table 4, QDT had a significant positive effect on ONS adherence (*β* = 0.162, *p* < 0.001), RHD had significant positive effects on ONS adherence (*β* = 0.228, *p* < 0.001), and medication beliefs had a significant positive effect on ONS adherence (*β* = 0.279, *p* < 0.001).

**TABLE 4:** The chain‐mediating regression equation (*N* = 505).

Regression equation		Overall ft. indices			Significance of regression coefficients		
Result variables	Predictive variables	*R*	*R* ^2^	*F*	*β*	95% CI	*t*
ONS adherence	QDT	0.604	0.364	18.677***	0.475	[0.402, 0.548]	12.824***
RHD	QDT	0.710	0.504	33.149***	0.660	[0.596, 0.725]	20.182***
Medication beliefs	QDT	0.729	0.532	34.633***	0.284	[0.199, 0.368]	6.582***
	RHD				0.454	[0.367, 0.540]	10.313***
ONS adherence	QDT	0.680	0.463	24.664***	0.162	[0.068, 0.257]	3.369***
	RHD				0.228	[0.125, 0.330]	4.371***
	Medication beliefs				0.279	[0.183, 0.374]	5.737***

Abbreviations: CI, confidence intervals; ONS, oral nutritional supplementation; QDT, quality of discharge teaching; RHD, readiness for hospital discharge.

*** *p* < 0.001.

### 3.6 The Construction of a Structural Equation Model

**FIGURE 3:**
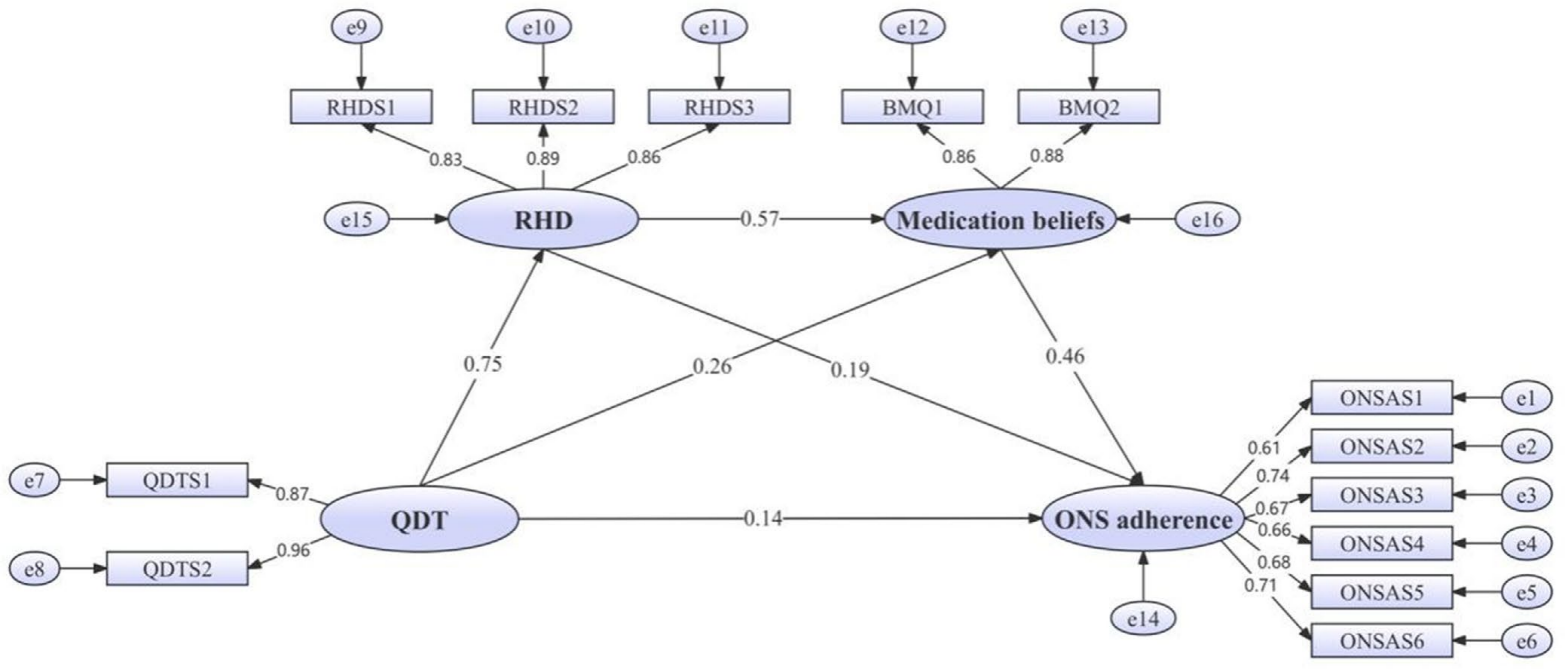
The chain mediation model diagram of QDT, RHD, medication beliefs and ONS adherence in postoperative patients with gastric cancer. BMQ, beliefs about medical questionnaire; ONS, oral nutritional supplementation; ONSAS, Oral Nutritional Supplementation Adherence Scale; QDT, quality of discharge teaching; QDTS, Quality of Discharge Teaching Scale; RHD, readiness for hospital discharge; RHDS, Readiness for Hospital Discharge Scale. [Colour figure can be viewed at wileyonlinelibrary.com]

The structural equation modelling for the mediation analysis was developed, using QDT as the independent variable, ONS adherence as the dependent variable, and RHD and medication beliefs serving as mediating variables. The results, as illustrated in Table 5, indicated a good data‐model fit. The diagram of the QDT, RHD, medication beliefs and ONS adherence for postoperative patients with GC is presented in Figure 3.

**TABLE 5:** Results of the model fit indicators (*N* = 505).

Classify	Absolute fitting indicator		Value‐added fit indicators			Simple fit indicator	
Sort	*χ* ^2^/df	RMSEA	GFI	TLI	CFI	PGFI	PNFI
Standard of judgement	< 5	< 0.05	> 0.9	> 0.9	> 0.9	> 0.5	> 0.5
Fit result	1.197	0.02	0.980	0.996	0.997	0.635	0.743

Abbreviations: CFI, Comparative Fit Index; df, degrees of freedom; GFI, Goodness of Fit Index; PGFI, Parsimony Goodness of Fit Index; PNFI, Parsimony Normed Fit Index; RMSEA, root mean square error of approximation; TLI, Tucker‐Lewis Index.

As shown in Table 6, the path coefficient from QDT to RHD (*β* = 0.747, *p* < 0.001), QDT to medication beliefs (*β* = 0.259, *p* < 0.001), RHD to medication beliefs (*β* = 0.574, *p* < 0.001), RHD to ONS adherence (*β* = 0.191, *p* = 0.021), medication beliefs to ONS adherence (*β* = 0.455, *p* < 0.001), and QDT to ONS adherence (*β* = 0.141, *p* = 0.035) were statistically significantly positive.

**TABLE 6:** Mediated effects test (*N* = 505).

Path	*β*	S.E.	C.R.	*p*	95% CI	
					Lower	Upper
RHD ← QDT	0.747	0.044	16.928	***	0.686	0.798
Medication beliefs ← QDT	0.259	0.144	4.34	***	0.138	0.385
Medication beliefs ← RHD	0.574	0.152	9.084	***	0.448	0.686
ONS adherence ← RHD	0.191	0.027	2.313	0.021	0.023	0.350
ONS adherence ← Medication beliefs	0.455	0.011	5.588	***	0.300	0.586
ONS adherence ← QDT	0.141	0.022	2.113	0.035	0.023	0.256

Abbreviations: *β*, beta; CI, confidence interval; C.R, critical ratio; ONS, oral nutritional supplementation; QDT, quality of discharge teaching; RHD, readiness for hospital discharge; S.E., standard error.

*** *p* < 0.001.

The Percentile Bootstrap method was further employed to accurately test the mediating effects of RHDS and medication beliefs in the relationship between QDT and ONS adherence. Table 7 illustrated that the direct effect of QDT on ONS adherence was 0.141 (95% CI 0.023, 0.256), supporting Hypothesis 1. Three pathways contributed to the total indirect effect, and the mediating effect of RHD between QDT and ONS adherence was 0.143 (95% CI 0.019, 0.268), supporting Hypothesis 2. Medication beliefs mediated the relationship between QDT and ONS adherence with an effect size of 0.118 (95% CI 0.057, 0.195), and Hypothesis 3 was verified. The chain‐mediating effect of RHD and medication beliefs on QDT and ONS adherence was 0.195 (95% CI 0.130, 0.278), thus Hypothesis 4 was verified. The 95% CI for each path did not include 0, indicating a statistically significant effect. These paths explained 23.9%, 19.8%, and 32.7% of the total effect (Shrout and Bolger 2002). None of the 95% CI include 0, suggesting statistically significant total, direct, and mediated effects.

**TABLE 7:** Mediated effects test (*N* = 505).

Effect	*β*	Bootstrapping 95% CI		*p*
		Lower	Upper	
QDT → RHD → ONS adherence	0.143	0.019	0.268	0.022
QDT → Medication beliefs → ONS adherence	0.118	0.057	0.195	< 0.001
QDT → RHD → Medication beliefs → ONS adherence	0.195	0.130	0.278	< 0.001
Direct effect	0.141	0.023	0.256	0.02
Total effect	0.597	0.538	0.651	0.001

Abbreviations: *β*, beta; CI, confidence interval; ONS, oral nutritional supplementation; QDT, quality of discharge teaching; RHD, readiness for hospital discharge; RME, relative mediating effect.

## 4 Discussion

This study explored the relationship between QDT, RHD, medication beliefs, and ONS adherence and developed a mediation model for postoperative patients with GC. The findings confirmed all four hypotheses, showing that QDT influences ONS adherence directly and indirectly through RHD and medication beliefs, as well as through the sequential mediation of RHD and medication beliefs. These findings could provide a nuanced understanding of the mechanisms driving ONS adherence and highlight theoretical and practical implications for clinical nursing practices.

The QDT score in this study was lower than recent findings (Guan and Feng 2023; Li et al. 2024; Yang et al. 2025), indicating suboptimal discharge teaching quality among GC patients. The comparatively lower score for teaching skills and effects than for teaching content acquired suggests a need to enhance the practical delivery of education. Previous research corroborates this by attributing ineffective teaching to nurses' workload and limited patient engagement time (Lyu et al. 2019). Hence, a multimodal and personalised approach should be implemented to elevate the teaching efficacy, supported by systemic reforms such as workflow optimisation and incentive mechanisms for nursing staff.

Our study identified poor ONS adherence (13.70 [SD = 5.32]) among participants, which was consistent with previous studies (Jiang et al. 2022; Su et al. 2024). Contributing factors may include lower education levels, limited financial resources, and social isolation, with 60.8% of participants having low or no income, and 83.8% having only junior high school education or less. A lower education level can hinder comprehension of discharge instructions, leading to poor adherence to treatment and self‐care protocols (Shahid et al. 2022). Limited financial resources may restrict patients' ability to purchase prescribed medications, access nutritious food, or attend follow‐up appointments (Rohatgi et al. 2021). Social isolation reduces the availability of emotional and practical support, which is crucial for managing health at home (National Academies of Sciences 2020). These barriers can collectively impair patients' ability to implement effective self‐care strategies, thereby increasing the risk of complications and hospital readmission. Literature supports that low health literacy and inadequate social support diminish treatment adherence (Gimeno‐Garcia et al. 2009; Seguy et al. 2020). In addition, 46.5% of the postoperative patients with GC recruited in this study were older, and 52.8% of the patients lived with their spouses or alone. According to the results of some surveys, post‐discharge patients with limited sources of social support tend to underdose or forget to take ONS, ultimately decreasing ONS adherence (Su et al. 2024; Wan, Yuan, et al. 2021). Thus, future intervention studies exploring strategies and measures are warranted to improve ONS adherence in patients with different conditions.

Moreover, our results showed a positive correlation between QDT and ONS adherence, indicating that the QDT significantly predicts ONS adherence in postoperative patients with GC, which is in line with the results of previous studies (Kee et al. 2018; Riester et al. 2022). The most common barriers in taking the ONS were bloating, early satiety, flavour or texture dislike, and diarrhoea (Liljeberg et al. 2024). Effective communication between nurses and patients facilitates the delivery of comprehensive discharge teaching. Upon hospital discharge, patients with richer self‐perceived knowledge and skills related to their disease and nutrition exhibit a clearer understanding of ONS and stronger trust in nurses (Jiang et al. 2022). Thus, prioritising discharge education can significantly enhance long‐term compliance.

This study used the structural equation modelling to verify the mediating effect of RHD on QDT and ONS adherence. The findings align with previous study (Yang et al. 2025), which reported that RHD partially mediated the relationship between QDT and adherence to self‐management behaviours. In addition, Zhang et al. observed that QDT not only had a direct impact on post‐discharge outcomes but also influenced them indirectly through RHD (Zhang et al. 2021). The QDT is the most significant predictor of RHD, and evaluating the effectiveness of discharge teaching through patients' self‐reports on the QDT can more accurately predict the perceived readiness and subsequent post‐discharge problems, including inadequate adherence (Mehraeen et al. 2022). Patients with higher perceived readiness tend to actively seek health‐related information after discharge, demonstrate a stronger ability to cope with and self‐manage challenges during the administration of ONS, and proactively seek medical help, which ultimately enhances their medication adherence (Yang et al. 2025). Conversely, inadequate readiness may hinder patients' ability to manage ONS independently, reducing their motivation and ultimately compromising adherence (Gray et al. 2021). These insights indicate the essential role of discharge teaching. Nurses must ensure patients are equipped with the necessary knowledge and skills for ONS self‐management, while nursing administrators should focus on improving discharge education quality and promoting patient readiness to enhance post‐discharge adherence.

This study revealed that medication beliefs partially mediated the relationship between the quality of QDT and ONS adherence in postoperative patients with GC. This result indicates that high‐quality discharge teaching not only directly influences adherence but also indirectly promotes adherence by strengthening patients' medication belief. Medication beliefs have long been recognised as a critical psychological determinant of treatment adherence across various health domains, including pharmacotherapy and rehabilitation (Naqvi et al. 2019). If postoperative patients with GC have higher QDT, they may have more positive perceptions of the medication necessity and less negative perceptions of the concerns, have more confidence to follow instructions of the ONS, gain more knowledge related to the management of the ONS, and adopt better ONS adherence behaviour to improve nutritional health in the long term (Lim et al. 2020; Su et al. 2024; Yarmohammadi et al. 2022). Previous studies have also found that nurses may communicate the necessity of medication more strongly, or the risks associated with non‐adherence during discharge teaching (Hardeman et al. 2014; Lin et al. 2022). But once patients leave the hospital, their concerns may increase, undermining adherence. This highlights the need for nurses to assess medication beliefs before discharge and provide targeted education to reinforce confidence in ONS, supporting long‐term nutritional recovery.

The results demonstrated that the relationship between QDT and ONS adherence is mediated by a sequential pathway involving RHD and medication beliefs. Specifically, RHD influences medication beliefs, which in turn affect adherence to ONS. This finding aligns with Melies' transition theory, when emphasises that postoperative patients who receive high‐quality discharge teaching are better prepared for discharge, reducing misconceptions and distrust regarding ONS (Lindquist et al. 2012). This enhanced preparation facilitates adherence to medical advice, improves ONS adherence, and ultimately leads to better post‐discharge outcomes (Zhang et al. 2021). Despite recent studies indicating the impact of interventions such as introducing fish oil to enrich ONS (Patursson et al. 2021; Schmidt et al. 2020) or delivering health education through the 5Ts for teach‐back (Wang et al. 2023), this study provides a novel theoretical framework that underscores the combined influence of RHD and medication beliefs. These findings suggest that interventions aimed at improving ONS adherence in postoperative GC patients should not only focus on product formulation or delivery methods but also prioritise discharge teaching quality and belief‐based counselling. Doing so could offer valuable strategies for clinical nurses and nursing managers seeking to enhance long‐term adherence outcomes in this population.

## 5 Limitations

There are some limitations in the present study. The first and main limitation of this study is the cross‐sectional design, which cannot draw causal inferences. Therefore, future longitudinal research designs are expected to further investigate these causal relationships among QDT, RHD, medication beliefs and ONS adherence. The second limitation is the reliance on a self‐report test to assess the four groups of variables, which may induce recall bias. Future investigations could include conducting interviews with patients and obtaining data from multiple channels, including in‐person, phone, and their caregivers. Thirdly, the limitation is its single‐centre setting. The study participants were recruited from three wards at one hospital in southeast China. Thus, future multi‐centre investigations should be conducted to enhance the generalisability and robustness of the findings. Fourthly, the follow‐up duration for ONS adherence is relatively short, lasting only 1 month, and future studies with longer follow‐up periods are warranted to confirm our findings. Lastly, ONS adherence is influenced by multiple factors, but only the influence of QDT on ONS adherence and the mechanism of RHD and medication beliefs were discussed in this study. Therefore, it is recommended to further investigate the mechanisms of factors associated with ONS adherence in postoperative patients with GC.

## 6 Conclusion

The present study indicated that the QDT influences ONS adherence in postoperative patients with GC, and RHD and medication beliefs mediated this effect. The study offers an understanding of the mechanisms behind the relationship between QDT and ONS adherence and provides a certain theoretical and practical direction for future research aimed at improving patients' QDT, RHD, medication beliefs, and ONS adherence in postoperative patients with GC.

## 7 Relevance to Clinical Practice

This present study used the structural equation modelling to shed light on the pathways of QDT, RHD, medication beliefs and ONS adherence, which offers a new viewpoint in the field of clinical nutrition and transitional care to improve the ONS adherence of postoperative patients with GC. With the advancement of the continuous nutritional nursing model, adherence and acceptance of ONS in cancer patients have become a widely discussed issue (Chen et al. 2022). Early intervention aimed at improving ONS adherence in postoperative patients with GC is of great practical significance. Therefore, future studies should focus on continuously exploring the nutritional requirements of postoperative patients with GC and developing personalised discharge teaching models and transitional care programmes to improve ONS adherence. This will provide a firm basis for the irrational application of ONS in postoperative patients with GC.

## Author Contributions

Jia Li, Xiuhong Yuan, and Fang Xiao conceived and drafted the protocol for this study. Fang Shen and Xiaoxue Chen designed and performed the experiments. Hong Yu analysed the data and prepared the figures. Fang Shen and Xiaoxue Chen wrote the manuscript. Jia Li, Xiuhong Yuan, and Fang Xiao revised the manuscript. Fang Shen, Xiaoxue Chen, and Hong Yu are the first authors. Jia Li, Xiuhong Yuan, and Fang Xiao were responsible for the study. All authors have reviewed and approved the final manuscript.

## Ethics Statement

Ethical approval for this study was obtained from the local Institutional Review Board (Sun Yat‐sen University Cancer Center; approval date: February 25, 2022; Reference: SL‐B2022‐091‐03).

## Consent

All participants provided written informed consent before engaging in the study.

## Conflicts of Interest

The authors declare no conflicts of interest.

## Supporting information


**Data S1.** Supporting Information.

## Data Availability

The datasets used and/or analysed during the current study are available from the corresponding author on reasonable request.
